# Spirometric reference equations for Cameroonians aged 4 to 89 years derived using lambda, mu, sigma (LMS) method

**DOI:** 10.1186/s12890-021-01705-1

**Published:** 2021-11-03

**Authors:** Eric Walter Pefura-Yone, Adamou Dodo Balkissou, Virginie Poka-Mayap, Amadou Djenabou, Massongo Massongo, Nguetsa Arsene Ofimboudem, Catherine Fanny Mayoh-Nguemfo, Antoinette Ghislaine Tsala, Halidou Hadjara, Francine Amougou

**Affiliations:** 1 Faculty of Medicine and Biomedical Sciences, University of Yaoundé 1, P.O Box 8340, Yaoundé, Cameroon; 2 Yaoundé Jamot Hospital, Yaoundé, Cameroon; 3 Association for the Promotion of Health, Research and Education (APHRE), Yaoundé, Cameroon; 4 Faculty of Medicine and Biomedical Sciences of Garoua, University of Ngaoundéré, Garoua, Cameroon; 5 Ngambé District Hospital, Ngambé, Cameroon; 6 Yaoundé Emergency and Resuscitation Center, Yaoundé, Cameroon; 7 Higher Institute of Medical Technology, Yaoundé, Cameroon

**Keywords:** Spirometry, Normal lung function, Reference values, Central Africa

## Abstract

**Background:**

Spirometric reference values are well known in several ethnic groups but the normative spirometric values of blacks living in Africa have been less studied. The purpose of this study is to establish normative spirometric equations from a representative population of Cameroonian children and adults and compare these equations with those developed by the Global Lung Initiative (GLI) and in Nigerians.

**Methods:**

Spirometric data from healthy Cameroonians aged 4–89 years randomly collected between 2014 and 2018 were used to derive reference equations using generalized additive model for location (mu), shape (lambda) and scale (sigma).

**Results:**

A total of 625 children and adolescents (290 males and 335 females) and 1152 adults (552 males and 600 females) were included in the study. The prediction equation for spirometric index was written as: M = Exp[a0 + a1*ln (Height) + a2*ln (Age) + Mspline, Mspline was age related spline contribution]. Applying the GLI standards for African Americans resulted in overall values greater than those found in our study for forced expiratory volume in 1s (FEV1) and forced vital capacity (FVC). These values were very close in children and adolescents while the values obtained with the GLI equations for African Americans were significantly higher in adults. FEV1/FVC ratio in our study was similar for adult males but lower in adult females (88% vs 85%, difference =  + 3.5%) when applying Nigerian standards.

**Conclusions:**

FEV1 and FVC of the Cameroonian infant and adolescent population are very close to those of black Americans. However, FEV1 and FVC of Cameroonian adults are significantly lower than those of black American adults. These equations should allow a more suitable interpretation of spirometry in the Cameroonian population.

**Supplementary Information:**

The online version contains supplementary material available at 10.1186/s12890-021-01705-1.

## Introduction

Lung function tests (LFTs) are of great value in the diagnosis, therapeutic management and prognosis of a broad range of respiratory diseases [1]. Values obtained during lung function tests are usually related to predicted or theoretical values. These predicted values are derived from the reference equations established from the values of the normal or “healthy” population. The normal spirometric values vary considerably according to ethnicity and the absence of spirometric reference values in a population thereby resulting in the misinterpretation of spirometric results [2–5]. International recommendations also require that the predicted values of pulmonary function tests used in a given population be those derived from the normal values of that population [6, 7]. Spirometric reference values are well known in several ethnic groups but the normative spirometric values of blacks living in Africa have been less studied [8]. Those that do exist were performed over 20 years ago or do not span all age groups [9–13]. Moreover, multi-ethnic equations covering ages 3–95 were published in 2012 but these equations do not include those of the black African population [8]. We did not find any validated reference spirometric equations in Cameroonian children and adults using robust methods. LFTs laboratories in sub-Saharan Africa commonly use normative values derived from the African American population without prior validation of these norms in the resident population of Africa. The application of these non-validated standards can be a source of misinterpretation of the spirometric values with a negative impact on patients’ care [3]. The objective of our study was to establish normative spirometric equations from a representative population of Cameroonian children and adults.

## Methods

### Design and participants

Data from cross-sectional surveys carried out from 2014 to 2018 in four regions of Cameroon were used. The subjects were recruited in the city of Yaoundé (urban area, Center region) from December 2014 to April 2015, in the health district of Bandjoun (semi-urban and rural area, Western region) from November 2015 to April 2016, in the city of Douala (urban area, Coastal region) from November 2016 to April 2017, in the city of Garoua (urban area, North region) and in Figuil (semi-urban and rural area, North region) from December 2017 to April 2018. Ethical clearance was obtained from the institutional ethics committees of the Faculty of Medicine and Biomedical Sciences of the University of Yaoundé 1, and of the Faculty of Medicine and Pharmaceutical Sciences of the University of Douala.

The sampling method used in this study has been published elsewhere [14, 15]. In summary, a 3-level stratified sampling method was applied in each recruitment area. At the 1st level, the enumerated areas corresponding to those used for national immunization days were selected by random sampling. At the 2nd level, households were selected by systematic sampling with variable sampling intervals depending on the size of each enumerated area. At the last level, all subjects from households selected at the second level and meeting both the inclusion and exclusion criteria were invited to participate in the study.

### Baseline data collection

Data was collected by final year medical student trained on the standardized questionnaire and the realization of spirometry. Demographic data including gender (male or female), age (calculated to the nearest month for children and adolescents) and ethnic group (Bantu, Sudano-Sahelian, mixed) were noted. Height and weight were measured for each subject and the body mass index (BMI) calculated as the ratio of the weight (kg) to the square of the height (m). "Healthy" subjects were selected using the American Thoracic Society (ATS) and National Heart and Lung -1978 respiratory questionnaire[16]. Subjects with the following conditions were excluded: recent respiratory symptoms (< 1 month), history of respiratory disease that may interfere with lung function [asthma, chronic bronchitis, chronic obstructive pulmonary disease (COPD)], tuberculosis and any other chronic respiratory disease), cardiovascular disease (heart failure, angina pectoris, myocardial infarction, severe uncontrolled hypertension), diabetes mellitus, stroke, smokers and ex-smokers, treatment with beta-blockers or bronchodilators, obesity (BMI ≥ 30 kg/m^2^ for adults, Z-score BMI > 2 for children and adolescents), underweight (BMI < 18.5 kg/m^2^ for adults, Z-score BMI < 2 for children and adolescents) and incorrect realization of the spirometric curves.

### Measure of spirometric parameters

Spirometry was performed in patients meeting the above inclusion criteria and without any contraindication. The methods for producing the flow-volume curve were those recommended by the American Thoracic Society/European Respiratory Society (ATS/ERS) in 2005[17]. The spirometric tests were carried out using a turbine pneumotachograph complying with2005-ATS/ERS standards including Spiro USB, Care fusion, Yorba Linda-USA or Spirobank II, MIR France, Langlade-France. The measurements were carried out under the supervision of a Pulmonologist who regularly performs and interprets LFTs.

All spirometric measurements were obtained from patients after a minimum of 15 min rest in a seated position, their backs straight with a nose clip to allow air movement only through the mouth. Full instructions on the realization of the test were clearly explained to each participant before the maneuver was performed.

All measurements were automatically corrected for body temperature and saturation pressure. The acceptability and reproducibility criteria recommended by the ATS/ERS were used[17]. Three to eight maneuvers were performed by each subject for the realization of the forced vital capacity (FVC) curve, with a resting period of at least one minute between each maneuver. The spirometric indices selected were: forced expiratory volume in 1 s (FEV1), forced vital capacity (FVC), FEV1/FVC ratio and forced mid-expiratory flow (FEF25–75%). The best FEV1 and FVC values among the three tests meeting the acceptability criteria were selected (maximum difference less than or equal to 5% or 150 ml compared to the other values). The maneuver with the highest FEV1 + FVC sum was kept for the derivation of the FEF25–75%.

### Data analysis

Our data were analyzed using R software version 4.0.3 [18]. The baseline characteristics and the spirometric parameters were analyzed separately for the male and female subjects and according to the different age groups (children: 4–12 years, adolescents: 13–18 years and adults: 19–89 years). Qualitative variables were summarized in terms of counts and proportions. The quantitative variables were summarized by their mean (standard deviation), median (25th–75th percentiles) and range. Scatterplots and box-plot (after discretization of age in 2-year increments and height in 5-year increments) were used to graphically represent the relationship between the spirometric and anthropometric parameters without and after logarithmic transformation. These graphical representations showed that the relationship between all the spirometric parameters and the anthropometric parameters was not linear regardless of the age group. Drawing inspiration from the latest studies on normative spirometric values and the complex effects of anthropometric parameters (explanatory factors, independent variables) on spirometric parameters (dependent variables), prediction models were developed using the generalized additive models for location, scale and shape, LMS imbedded in GAMLSS. Spirometric parameters (FEV1, FVC, FEV1/FVC, FEF25–75%) can be characterized by their mean (location, M or mu), coefficient of variation (scale, S or sigma) and their skewness coefficient (shape, L or lambda). These characteristics are summarized by the acronym LMS. The prediction analyses were done by the GAMLSS package of the R software [19]. The complex effects of the explanatory variables on the dependent variable can thus be modeled in a smooth and non-linear way using splines and thus make it possible to obtain a smooth modeling over all age groups. We used the Box-Cox-Cole-Green distribution described by Cole et al. to estimate the best prediction model of the spirometric parameters while avoiding over-modeling [20]. Thus, the models with the LMS indices giving the smallest Schwarz Bayesian criterion (SBC) were selected for each spirometric parameter separately in male and female subjects. The general form of the equation for each spirometric parameter was of the form:

Y = a + b * height + c * age + spline (spline is an age-specific contribution from the spline function). The best models were obtained after the logarithmic transformation of the parameters, thus giving an equation of the final form: ln (Y) = a1 + a2 * ln (height) + a3 * ln (age) + spline or Y = exp (a1 + a2 * ln (height) + a3 * ln (age) + spline). For ages whose splines were not obtained directly from the table of spline values, a linear interpolation was carried out according to the formula:

Xspline (age) = [(age2—age) * Xspline (age1) + (age − age1) * Xspline (age2)]/(age2 − age1); X: L, M or S; Xspline (age): spline corresponding to a given age, age2: upper limit of the interpolation age, age1: lower limit of the interpolation age. The lower limit of normal (LLN) corresponds to the 5th percentile of M. The splines and the corresponding coefficients as well as the formulas for calculating the predicted values were recorded in the lookup tables. The following formula was used to calculate deviation indices (% difference): % difference = (predicted parameter according to other references—predicted parameter in our equation)/ predicted parameter in our equation.

## Results

### Study population

The reasons for exclusion are shown in Table 1. In children and adolescents (n = 1098), the main reasons for exclusion were unacceptable spirometric maneuvers (137 cases, 12.5%) and the existence of respiratory symptoms (104 cases, 9.5%) or a history of respiratory diseases (72 cases, 6.6%). In adults (n = 5055), the main reasons for exclusion were obesity (1382 cases, 27.3%), unacceptable maneuvers (935 cases, 18.5%), tobacco smoking (844 cases, 16.7%) and history of respiratory symptoms (550 cases, 10.9%). A total of 625 children and adolescents (290 males and 335 females) and 1152 adults (552 males and 600 females) were included in the final analysis.

**Table.1:** Reasons for exclusion of participants

Reasons	Children and adolescents (4–18 years) n = 1098 (%)	Adults (≥ 19 years) n = 5055 (%)
Unacceptable maneuvers	137 (12.5)	935 (18.5)
Respiratory symptoms	104 (9.5)	550 (10.9)
Chronic respiratory diseases	72 (6.6)	510 (10.1)
Obesity	32 (2.9)	1382 (27.3)
Tobacco smoking	54(4.9)	844(16.7)
Others	86(7.8)	211 (4.2)

The general characteristics of subjects included are shown in Table 2. The ages ranged from 4.95 to 88 years for male participants and from 4.45 to 89 years for female participants. The height of the participants ranged from 103 to 182 cm for male children and adolescents and from 104 to 175 cm for female children. In adults, the height ranged from 150 to 196 cm for males and 143 to 188 cm for females.

**Table.2:** general characteristics of study population

Parameters	Children (4–12 years)	Adolescents (13–18 years)	Adults (≥ 19 years)
*Males, n*	179	111	552
Age, years			
Range	4.95–11.95	12.01–18.73	19.01–87.16
Mean (SD)	8.91 (1.77)	14.63 (1.73)	37.24 (15.45)
Median (25–75th percentiles)	9.05 (7.58–10.29)	14.56 (13.16–15.79)	31.84 (24.89–46.07)
Height, cm			
Range	103–161	134–182	150–196
Mean (SD)	131.65 (10.82)	160.04 (10.29)	172.13 (7.17)
Median (25–75th percentiles)	132 (124–139)	160 (153–166.5)	172 (168–176)
Ethnicity			
Bantu, n (%)	168 (93.8)	104 (93.7)	415 (75.2)
Soudano-sahelian, n (%)	11 (6.2)	7 (6.3)	105 (19.0)
Mixed	–	–	32 (5.8)
FEV1, L			
Range	0.84–3.13	1.58–4.02	1.05–5.91
Mean (SD)	1.51 (0.39)	2.72 (0.61)	3.26 (0.81)
Median (25–75th percentiles)	1.50 (1.25–1.74)	2.69 (2.25–3.15)	3.29 (2.78–3.75)
FVC, L			
Range	0.86–3.34	1.76–4.58	1.28–7.51
Mean (SD)	1.65 (0.45)	3.07 (0.70)	3.82 (0.90)
Median (25–75th percentiles)	1.61 (1.34–1.93)	2.93 (2.56–3.61)	3.79 (3.32–4.40)
FEV1/FVC			
Range	0.75–1	0.77–0.99	0.65–1
Mean (SD)	0.92	0.89 (0.05)	0.85 (0.07)
Median (25–75th percentiles)	0.93 (0.88–0.97)	0.89 (0.86–0.92)	0.86 (0.81–0.90)
FEF25-75%, L/S			
Range	0.93–4.90	1.71–5.06	0.84–10.44
Mean (SD)	2.06 (0.64)	3.23(0.82)	4.52(1.75)
Median (25–75th percentiles)	2.03 (1.62–2.44)	3.22(2.61–3.94)	4.24(3.31–5.46)
*Females, n*	217	118	600
Age, years			
Range	4.45–11.98	12.02–18.67	19–88.13
Mean (SD)	8.79(1.78)	15.33 (2.00)	38.09 (15.54)
Median (25–75th percentiles)	8.89(7.40–10.16)	15.4 (13.40–17.24)	33.20 (24.67–49.31)
Height, cm			
Range	104–174	140–175	143–188
Mean (SD)	132.20 (12.41)	158.30 (7.13)	162.43 (6.62)
Median (25–75th percentiles)	130 (124–140)	158 (154–163)	162 (158–167)
Ethnicity			
Bantu, n (%)	203(93.5)	106 (89.8)	481 (80.2)
Soudano-sahelian; n (%)	14(6.5)	12 (10.2)	68 (11.3)
Mixed	–	–	51 (8.5)
FEV1, L			
Range	0.65–3.20	1.64–3.40	1.01–4.58
Mean (SD)	1.48 (0.44)	2.56 (0.40)	2.42 (0.60)
Median (25–75th percentiles)	1.42 (1.18–1.74)	2.55 (2.31–2.81)	2.46 (1.98–2.80)
FVC, L			
Range	0.70–3.58	1.82–3.81	1.13–5.19
Mean (SD)	1.62 (0.49)	2.81 (0.45)	2.83 (0.66)
Median (25–75th percentiles)	1.54 (1.27–1.89)	2.83 (2.50–3.13)	2.82 (2.40–3.24)
FEV1/FVC			
Range	0.79–1	0.78–1	0.65–1
Mean (SD)	0.92 (0.05)	0.91 (0.07)	0.85 (0.07)
Median (25–75th percentiles)	0.93 (0.88–0.97)	0.90 (0.87–0.94)	0.86 (0.80–0.91)
FEF25-75%, L/S			
Range	0.74–4.67	1.62–4.91	1–8.07
Mean (SD)	0.92 (0.88–0.97)	3.21 (0.73)	3.57 (1.27)
Median (25–75th percentiles)	0.93 (0.88–0.97)	3.21 (2.69–3.68)	3.42 (2.64–4.35)

FEV1, forced expiratory volume in 1s (FEV1); FVC, forced vital capacity; FEF25-75%, forced mid-expiratory flow, SD: standard deviation

### Relationship between spirometric indices and anthropometric parameters

**Fig. 1:**
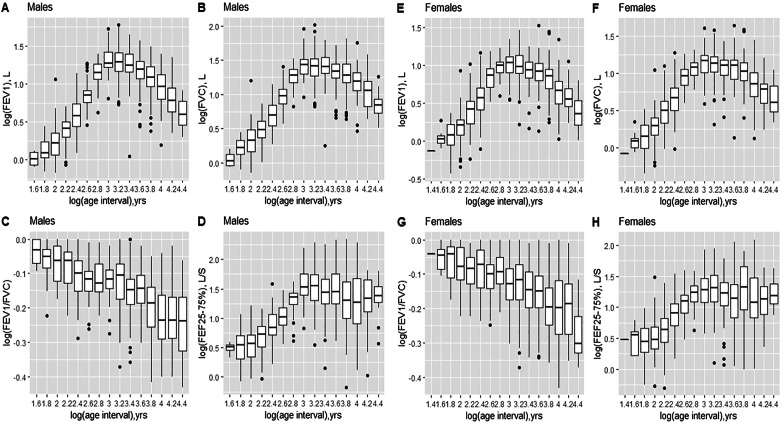
Relationship between spirometric indices and age in males** (A, B, C, D)** and females** (E, F, G, H)** after natural logarithmic transformation. FEV1, forced expiratory volume in 1 s (FEV1); FVC, forced vital capacity; FEF25-75%, forced mid-expiratory flow

**Fig. 2:**
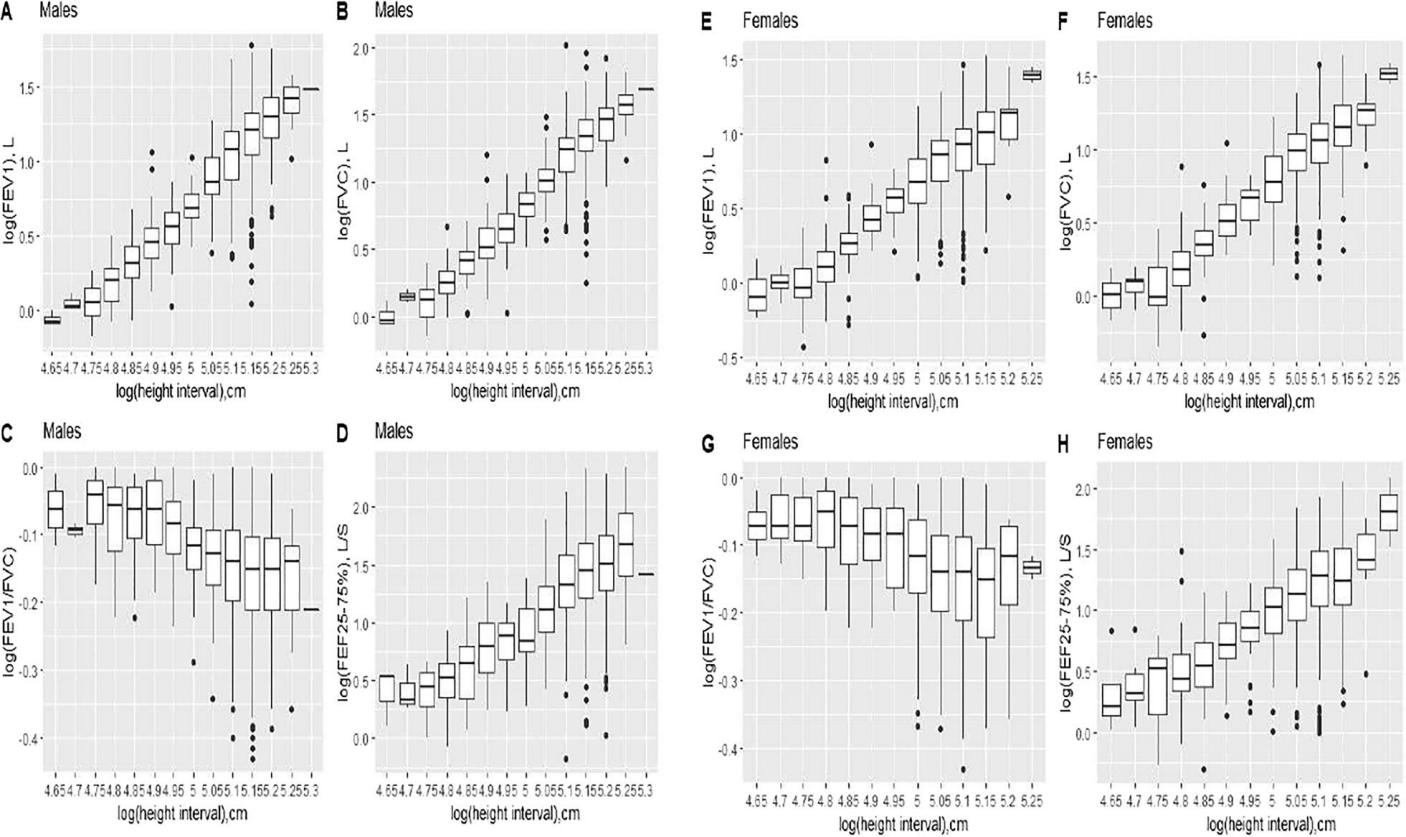
Relationship between spirometric indices and height in males** (A, B, C, D)** and females** (E, F, G, H)** after natural logarithmic transformation. FEV1, forced expiratory volume in 1 s (FEV1); FVC, forced vital capacity; FEF25-75%, forced mid-expiratory flow

The relationship between spirometric indices and age is shown in Supplementary Fig. S1 and that showing the relationship between spirometric indices and height in Supplementary Fig. S2. Graphical representations using logarithmic transformation of spirometric indices and anthropometric parameters show that the relationship between spirometric indices and anthropometric parameters is not linear regardless of the age group considered (Figs. 1 and 2).

FEV1, FVC, and FEF25-75% increase with age through adolescence and then decrease from 25 years. For the FEV1/FVC ratio there is a gradual decrease from childhood to adulthood. In adolescents and young adults (13–25 years), the amplitude of the increase or decrease is less marked compared to other age groups (< 13 years and > 25 years), Additional file 1. These three indices: FEV1, FVC and FEF25-75% also increase nonlinearly with height (Additional file 2). The relationship between FEV1/FVC ratio and height is more complex especially in female subjects with a gradual decrease to 154 cm, then an increase to 158 cm, followed by a steady decrease to maximum height (Additional file 2).

### Prediction models

The prediction equations for all spirometric indices were written as:

M = exp (a0 + a1 * ln (H) + a2 * ln (A) + Mspline) [M = mu = spirometric index, H = height in cm, A = age in years, a0 = constant, a1 and a2 = coefficients associated with height and age, Mspline = age spline for the variable M or mu].

The scale (sigma) was obtained by the equation: S = sigma = exp (p0 + p1 * ln (Age) + Sspline) [p0 = constant, p1 = coefficient associated with age, Sspline = spline for the variability).

The skewness (nu) was obtained by the equation: L = nu = q0 + q1 * ln (Age) + Lspline [q0 = constant, q1 = coefficient associated with age, Lspline = spline for the skewness).

LLN for each spirometric index was given by the formula LLN = exp (ln (1 − 1.645 * L * S)/L + ln (M)).

The Z-score for each index was written as: Z-score = [(measured value / M)^L^ − 1] / L * S.

The percentage of the predicted value, % predicted was written as: % predicted = (measured value / M) * 100. The intercepts and coefficients for each spirometric index are presented in Excel format separately for male and female subjects in Additional file 3. The prediction equations are summarized in Table 3. The spline reference tables (lookup tables) are also presented in these files (Additional file 3). For ages that do not have an exact value of splines, a linear interpolation must be done to obtain the spline to use as indicated in the data analysis section. The SPSS syntaxes used to calculate all the values are available as zip Additional file 4.

**Table.3:** Lamba, Mu and Sigma (LMS) equations for spirometric indices of Cameroonian

Spirometric indices	Males	Females
FEV1, L		
Mu	Exp[− 9.47 + 2.03*ln(Height) + 0.05*ln(Age) + Mspline]	Exp[− 9.28 + 2.02*ln(Height) − 0.04*ln(Age) + Mspline]
Sigma	Exp[− 2.25 + 0.13*ln(Age) + Sspline]	Exp[− 2.14 + 0.11*ln(Age) + Sspline]
Lambda	− 3.50 + 1.36*ln(Age) + Lspline	1
FVC, L		
Mu	Exp[− 10.10 + 2.15*ln(Height) + 0.09*ln(Age) + Mspline]	Exp[-9.60 + 2.08*ln(Height) + 0.01*ln(Age) + Mspline]
Sigma	Exp[− 2.13 + 0.10*ln(Age) + Sspline]	Exp[− 2.05 + 0.09*ln(Age) + Sspline]
Lambda	− 2.59 + 1.01*ln(Age) + Lspline	1
FEV1/FVC		
Mu	Exp[0.58 − 0.11*ln(Height) − 0.05*ln(Age) + Mspline]	Exp[0.29 – 0.05*ln(Height) – 0.06*ln(Age) + Mspline]
Sigma	Exp[− 3.41 + 0.22*ln(Age) + Sspline]	Exp[− 3.50 + 0.27*ln(Age) + Sspline]
Lambda	1	1
FEF25-75%		
Mu	Exp[− 7.34 + 1.60*ln(Height) + 0.15*ln(Age) + Mspline]	Exp[− 8.30 + 1.81*ln(Height) + 0.09*ln(Age) + Mspline]
Sigma	Exp[− 2.14 + 0.32*ln(Age) + Sspline]	Exp[− 1.77 + 0.19*ln(Age) + Sspline]
Lambda	0.02 + 0.08*ln(Age) + Lspline	− 0.54 + 0.32*ln(Age) + Lspline

Xspline: age spline for Mu, Sigma or Lamda

### Models comparison

**Fig. 3:**
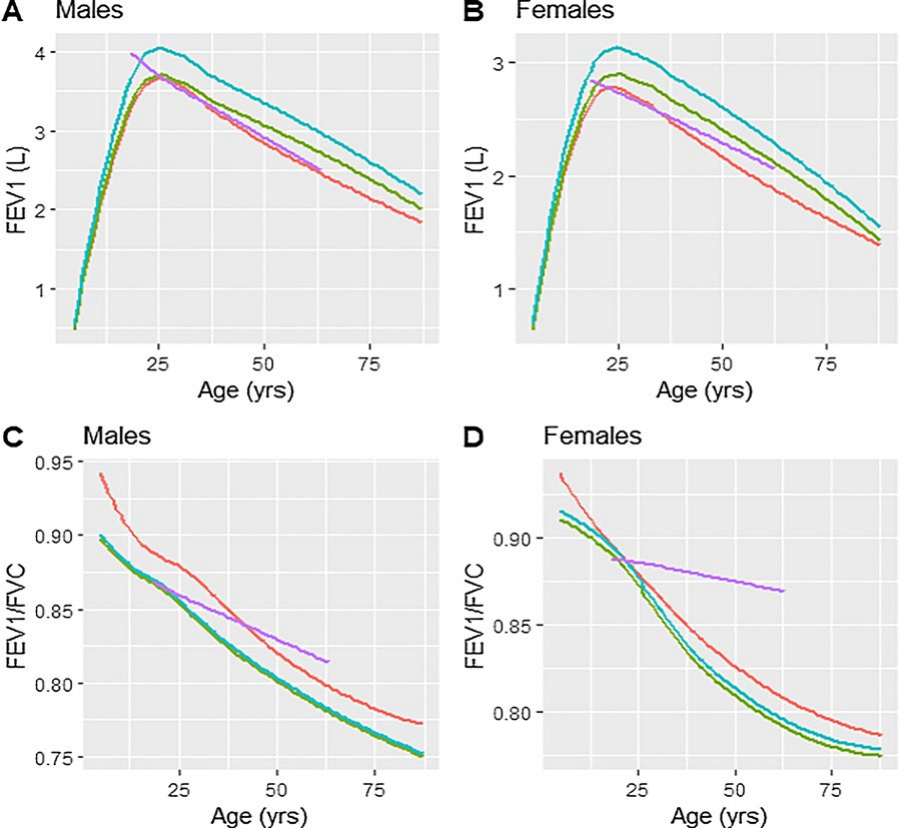
Comparison of our prediction equations of FEV1 and FEV1/FVC ratio with other reference equations using mean height for each age group. FEV1, forced expiratory volume in 1 s (FEV1); FVC, forced vital capacity. Equations 1 orange line, 2 green line, 3 blue line, 4 violet line. 1, current study; 2, GLI 2012 African Americans; 3, GLI 2012 other ethnic groups; 4, Fawibe et al. Nigerian equations

The spirometric reference equations derived from our sample were compared with the equations derived from GLI for African Americans, GLI for other ethnic groups and Nigerians, Fig. 3 [8, 21].

Table 4 shows the variation of the spirometric indices obtained with the equations developed in our study compared to the values obtained with the equations derived from other studies using the mean height for each age group.

**Table.4:** Deviation indices (% difference) comparing other spirometric references to our prediction equation

Reference equations	Males				Females			
	Mean FEV1, L (% difference)	Mean FVC, L (% difference)	Mean FEV1/FVC (% difference)	FEF25-75%, L/S (% difference)	Mean FEV1, L (% difference)	Mean FVC, L (% difference)	Mean FEV1/FVC (% difference)	FEF25-75%, L/S (% difference)
**Children (4–12 years)**								
Current study	1.49	1.63	0.92	2.00	1.48	1.62	0.92	1.99
GLI for African American	1.49 (0%)	1.70 (+ 4.3)	0.89 (− -3.3%)	1.82 (− 9.0%)	1.54 (+ 4.1%)	1.72 (+ 6.2%)	0.90 (− 2.2%)	2.04 (+ 2.5%)
GLI for other ethnic group	1.63 (+ 9.4%)	1.85 (+ 13.5%)	0.89 (− 3.3%)	1.98 (− 1.0%)	1.66 (+ 12.2%)	1.85 (+ 14.2%)	0.91 (− 1.1%)	2.19 (+ 10.1%)
Nigerian	/	/	/	/	/	/	/	/
**Adolescents (13–18 years)**								
Current study	2.70	3.04	0.89	3.23	2.51	2.79	0.90	3.20
GLI for African American	2.66 (− 1.5%)	3.04 (0%)	0.88 (− 1.1%)	3.12 (− 3.4%)	2.60 (+ 3.6%)	2.91 (+ 4.3%)	0.90 (0%)	3.24 (+ 1.3%)
GLI for other ethnic group	2.90 (+ 7.4%)	3.31 (+ 8.9%)	0.88 (− 1.1%)	3.39 (+ 5.0%)	2.81 (+ 12.0%)	3.12 (+ 11.8%)	0.90 (0%)	3.48 (+ 8.8%)
Nigerian	3.90 (+ 44.4%)	4.51 (+ 48.4%)	0.87 (-2.2%)	5.80 (+ 79.6%)	2.83 (+ 12.7%)	3.20 (+ 14.7%)	0.89 (− 1.1%)	4.96 (+ 55%)
**Adults (19–88 years)**								
Current study	3.27	3.81	0.85	4.30	2.42	2.83	0.85	3.48
GLI for African American	3.40 (+ 4.0%)	4.11 (+ 7.9%)	0.83(-2.4%)	3.48 (− 19.1%)	2.59 (+ 7.0%)	3.09 (+ 9.2%)	0.84 (− 1.2%)	2.87 (− 17.5%)
GLI for other ethnic group	3.71 (+ 13.5%)	4.47 (+ 17.3%)	0.83 (− 2.4%)	3.79 (− 11.9%)	2.80 (+ 15.7%)	3.32 (+ 17.3%)	0.85 (0%)	3.07 (− 11.8%)
Nigerian	3.45 (+ 5.2%)	4.02 (+ 5.5%)	0.85 (0%)	5.01 (+ 16.5%)	2.54 (+ 5.0%)	2.85 (+ 0.7%)	0.88 (+ 3.5%)	4.32 (+ 24.1%)

FEV1, forced expiratory volume in 1 s (FEV1); FVC, forced vital capacity; FEF25-75%, forced mid-expiratory flow

#### FEV1 and FVC

Applying the GLI standards for African Americans resulted in overall values greater than those found when applying the equations derived from our study for FEV1 and FVC. These values are very close in children and adolescents (the difference not exceeding + 5% except in female children in whom the difference was + 6.2% for FVC), while the values obtained with the GLI equations for African Americans [8] were significantly higher in adults, with an average difference reaching + 9.2% for FVC in adult women.

The Nigerian equations of Fawibe et al. [21] gave a great difference for FEV1 and FVC in adolescents (+ 44.4% and + 48.4%, respectively in adolescent males and females). On the other hand, in adults, the application of these Nigerian standards gave a difference of + 5.2% for FEV1 in men and + 5% for FEV1 in women.

#### FEV1/FVC ratio

The predicted FEV1/FVC ratio derived from our study was slightly higher in all age groups compared to that derived by applying the GLI equations for African Americans or for other ethnic groups (difference ranging from -3.3% in male children to 0% for adolescent females) [8]. The FEV1/FVC ratio predicted in our study was similar for adult males but lower in adult females (88% vs 85%, difference =  + 3.5%) when applying Nigerian standards [21].

#### FEF25-75%

For children and adolescents, the GLI equations and those derived in our study gave very close values for the FEF25-75% in most cases (difference ranging from − 3.4% to + 2.5%) except for male children (difference =− 9.0%). The FEF25-75% predicted by our equations was significantly higher than that predicted by the GLI equations for African Americans in adult males (difference = − 19.1%) and in adult females (difference = − 17.5%)[8]. On the other hand, the FEF25–75% predicted by the Nigerian equations was significantly higher than that predicted by our equations (difference of + 24.1% in adult women) [21].

## Discussion

To the best of our knowledge, this is one of the first studies which estimate normal spirometric parameters in the black African population living in Central Africa and spanning all age groups. We used a robust statistical method to take into account the complex non-linear relationship between spirometric parameters and the anthropometric indices explaining the normal spirometric values. The non-linear relationship between the spirometric and anthropometric parameters is particularly complex between 13 and 25 years old (adolescence and early adulthood). There is therefore the need of a statistical method which will model this complexity, thereby rendering the estimation of spirometric parameters smooth around pivotal periods of childhood-adolescence and adolescence-adulthood transition. The other major information resulting from this study is that: the Cameroonian normative spirometric values of FEV1 and FVC for children are close to those of the GLI for African Americans (difference of less than + 6.2%) but these spirometric values for adult subjects are significantly lower than those of African American adults (difference reaching + 9.2%); The FEV1/FVC ratio of Cameroonian adults is higher than that of African Americans, and the greatest variability was found for the comparison of the FEF25–75% between the different equations.

Our equations are applicable in the Cameroonian population of age varying from 4 to 88 years for males and from 4 to 89 years for females. Very few studies are available in Africa for spirometric equations for children aged 4 to 13 years as well as for subjects over 70 years of age. For example, in a recent study carried out in Nigeria in a population close to the Cameroonian population, the authors only included subjects aged 18 to 65 years [21]. The availability of normal spirometric equations resulting from the standards of the Cameroonian population will allow a better adapted interpretation of the LFTs in our laboratories currently applying the GLI or other standards.

Comparison of our FEV1 and FVC equations with those of the GLI for African Americans showed small differences in children not exceeding + 6.2% but in adults the difference reached + 9.2%. It is commonly believed that the differences in parameters of lung function observed between ethnic groups are related to genetic factors, environmental conditions, nutritional factors and physical activity [22]. There are complex interactions with non-proportional differences across all age groups as found in our study[8]. The increase in the difference in adulthood found in the FEV1 and FVC norms when comparing our equations with those of the GLI for African Americans probably indicates the existence of factors acting from adolescence on the reduction of spirometric parameters. Non-genetic factors such as physical activities, nutritional status and environmental factors could be responsible for the reduction of normative values in black subjects living in Africa.

The FEV1/FVC ratio in males is slightly higher in all age groups than in black Americans or other ethnic groups. For female subjects, the same pattern is found except for adolescent subjects in whom this ratio was similar. It is commonly believed that the FEV1/FVC ratio varies little between ethnic groups [8] but some studies report significantly higher FEV1/FVC values in blacks living in Africa and in Asian groups [10, 21, 23, 24]. This variability of the FEV1/FVC ratio can be explained by a non-proportional variation of FEV1 and FVC in the different ethnic groups.

The FEF25-75% is usually reported to have the greatest variability compared to other spirometric indices [8]. Comparing our equations with those of African Americans also shows greater variability for the FEF25-75%. Nevertheless, the increasing use of the Z-score in the interpretation of normative spirometric values should allow a more accurate use of this parameter and an improvement in the screening of distal airway obstruction based on the FEF25-75%.

The main limitations of our study are the small number of subjects aged 80 years and above and its cross-sectional nature. The cross-sectional nature of our study does not consider individual variations in the spirometric parameters over time, thereby making it difficult to predict the individual trajectory of respiratory function throughout life. The subjects included in this study were recruited during different years from 2014 to 2018. It is possible that the spirometric values be influenced by the secular evolution of the participants. However the period of inclusion is relatively short (4 years) to induce a notable secular modification of the spirometric parameters. Moreover, in a large study published in 2011 by Quanjer et al., no secular modification of the main spirometric parameters (FEV1, FVC, FEV1/FVC ratio) was found by collating data from 30 spirometric datasets carried out between 1978 and 2009[25].

In this study, we used two brands of spirometers (Spiro USB, Care fusion, Yorba Linda-USA or Spirobank II, MIR France, Langlade-France) based on the same technology. The two brands use a turbine pneumotachograph complying with the ATS/ERS standards of 2005 with a precision on the measured volume of ± 3% for the Care fusion spiro USB and of ± 2.5% for the MIR Spirobank II. For these reasons we believe that the spirometric measurements obtained were not influenced by the type of spirometer.

The use of the GAMLSS method for the prediction of normal values in our study makes it possible to take into account the variations of the values for each 2-years increment and to make the estimation of the spirometric indices more robust compared to the use of classic linear models.

## Conclusions

In this study, we presented the prediction equations of the spirometric parameters of the Cameroonian population aged 4 to 89 years using the GAMLSS statistical method allowing to model each of the spirometric parameters over the entire age group. The FEV1 and FVC of the Cameroonian population are very close to those of black American children and adolescents. On the contrary, the FEV1 and FVC of Cameroonian adults are significantly lower than those of black American adults. These equations should allow a more suitable interpretation of spirometry in the Cameroonian population. Data from other African populations should also make it possible to study the variation in lung function in different regions of sub-Saharan Africa.

## Supplementary Information

**Additional file 1**. Scatterplots showing relationship between spirometric indices and age in males (A, B, C, D) and females (E, F, G, H). FEV1, forced expiratory volume in 1s (FEV1); FVC, forced vital capacity; FEF25-75%, forced mid-expiratory flow

**Additional file 2**. Scatterplots showing relationship between spirometric indices and height in males (A, B, C, D) and females (E, F, G, H). FEV1, forced expiratory volume in 1s (FEV1); FVC, forced vital capacity; FEF25-75%, forced mid-expiratory flow

**Additional file 3**. Lookup tables for splines and coefficients to derive normal spirometric values for Cameroonian aged 4 to 89 years old.

**Additional file 4**. SPSS syntaxes for calculation of normal spirometric values for Cameroonian aged 4 to 89 years old.

## Data Availability

The datasets used and/or analyzed during the current study are available from the corresponding author on reasonable request.

## Abbreviations

ATS: American Thoracic Society
BMI: Body mass index
COPD: Chronic obstructive pulmonary disease
ERS: European Respiratory Society
FEF25-75%: Forced mid-expiratory flow
FEV1: Forced expiratory volume in 1s
FVC: Forced vital capacity
GAMLSS: Generalized additive model for location, scale and shape
GLI: Global Lung Initiative
LFTs: Lung function tests
LLN: Lower limit of normal
