# Supplementary figures and images for: Spirometric reference equations for Cameroonians aged 4 to 89 years derived using lambda, mu, sigma (LMS) method
Source: BMC Pulm Med. 2021 Nov 3;21:344. doi: 10.1186/s12890-021-01705-1 (PMC8565080; doi:10.1186/s12890-021-01705-1)

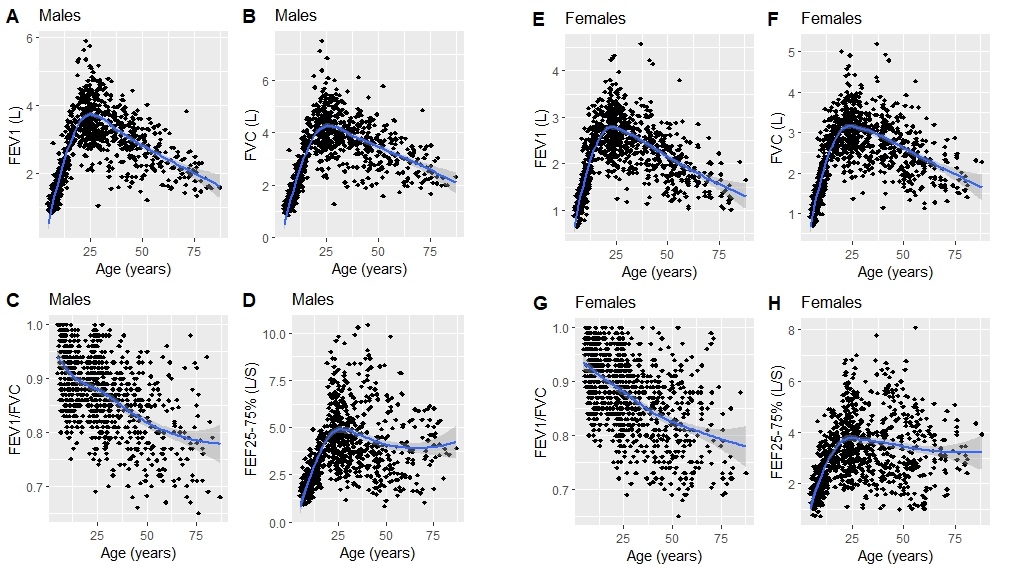

Supplement: Supplementary file 1 — Additional file 1. Scatterplots showing relationship between spirometric indices and age in males (A, B, C, D) and females (E, F, G, H). FEV1, forced expiratory volume in 1s (FEV1); FVC, forced vital capacity; FEF25-75%, forced mid-expiratory flow [file 12890_2021_1705_MOESM1_ESM.jpg]

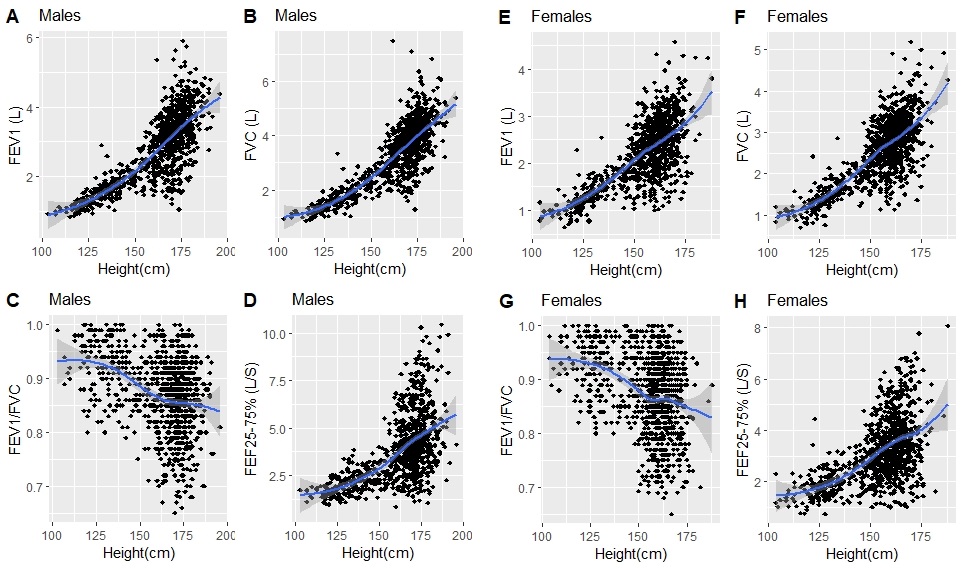

Supplement: Supplementary file 2 — Additional file 2. Scatterplots showing relationship between spirometric indices and height in males (A, B, C, D) and females (E, F, G, H). FEV1, forced expiratory volume in 1s (FEV1); FVC, forced vital capacity; FEF25-75%, forced mid-expiratory flow [file 12890_2021_1705_MOESM2_ESM.jpg]
